# γ‐aminobutyric acid measurement in the human brain at 7 T: Short echo‐time or Mescher–Garwood editing

**DOI:** 10.1002/nbm.4706

**Published:** 2022-02-18

**Authors:** Song‐I Lim, Lijing Xin

**Affiliations:** ^1^ Laboratory of Functional and Metabolic Imaging École polytechnique fédérale de Lausanne (EPFL) Lausanne Switzerland; ^2^ CIBM Center for Biomedical Imaging Switzerland; ^3^ Animal Imaging and Technology Ecole Polytechnique Fédérale de Lausanne (EPFL) Lausanne Switzerland

**Keywords:** GABA, MEGA, MRS, reproducibility, short TE, sSPECIAL

## Abstract

The purposes of the current study were to introduce a Mescher–Garwood (MEGA) semi‐adiabatic spin‐echo full‐intensity localization (MEGA‐sSPECIAL) sequence with macromolecule (MM) subtraction and to compare the test–retest reproducibility of γ‐aminobutyric acid (GABA) measurements at 7 T using the sSPECIAL and MEGA‐sSPECIAL sequences. The MEGA‐sSPECIAL editing scheme using asymmetric adiabatic and highly selective Gaussian pulses was used to compare its GABA measurement reproducibility with that of short echo‐time (TE) sSPECIAL. Proton magnetic resonance spectra were acquired in the motor cortex (M1) and medial prefrontal cortex (mPFC) using the sSPECIAL (TR/TE = 4000/16 ms) and MEGA‐sSPECIAL sequences (TR/TE = 4000/80 ms). The metabolites were quantified using LCModel with unsuppressed water spectra. The concentrations are reported in institutional units. The test–retest reproducibility was evaluated by scanning each subject twice. Between‐session reproducibility was assessed using coefficients of variation (CVs), Pearson's *r* correlation coefficients, and intraclass correlation coefficients (ICCs). Intersequence agreement was evaluated using Pearson's *r* correlation coefficients and Bland–Altman plots. Regarding GABA measurements by sSPECIAL, the GABA concentrations were 0.92 ± 0.31 (IU) in the M1 and 1.56 ± 0.49 (IU) in the mPFC. This demonstrated strong between‐session correlation across both regions (*r* = 0.81, *p* < 0.01; ICC = 0.82). The CVs between the two scans were 21.8% in the M1 and 10.2% in the mPFC. On the other hand, the GABA measurements by MEGA‐sSPECIAL were 0.52 ± 0.04 (IU) in the M1 and 1.04 ± 0.24 (IU) in the mPFC. MEGA‐sSPECIAL demonstrated strong between‐session correlation across the two regions (*r* = 0.98, *p* < 0.001; ICC = 0.98) and lower CVs than sSPECIAL, providing 4.1% in the M1 and 5.8% in the mPFC. The MEGA‐editing method showed better reproducibility of GABA measurements in both brain regions compared with the short‐TE sSPECIAL method. Thus it is a more sensitive method with which to detect small changes in areas with low GABA concentrations. In GABA‐rich brain regions, GABA measurements can be achieved reproducibly using both methods.

## Graphical abstract

(A‐D) illustrate overlay spectra from the first and second sessions acquired using sSPECIAL at short‐TE and MEGA‐sSPECIAL in the M1 and mPFC. (E and F) show coefficients of variation of each measurement method depending on the number of averages.

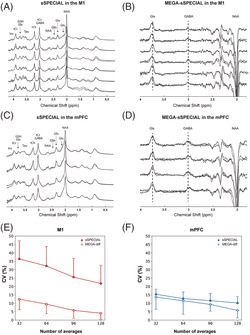

**Abbreviations used**
Ala: alanine
Asc: ascorbate
Asp: aspartate
Cr: creatine
CV: coefficient of variation
FWHM: full width half maximum
GABA: γ‐aminobutyric acid
Glc: glucose
Gln: glutamine
Glu: glutamate
Gly: glycine
GPC: glycerophosphorylcholine
GSH: glutathione
ICC: intraclass correlation coefficient
IU: institutional unit
Lac: lactate
LASER: localized by adiabatic selective refocusing
Lys: lysine
MEGA: Mescher–Garwood
MMs: macromolecules
mPFC: medial prefrontal cortex
M1: motor cortex
mIns: myo‐inositol
NAA: N‐acetylaspartic acid
NAAG: N‐acetylaspartylglutamate
PCho: phosphorylcholine
PCr: phosphocreatine
PE: phosphorylethanolamine
PRESS: point‐resolved spectroscopy sequence
^1^H‐MRS: proton magnetic resonance spectroscopy
sIns: scyllo‐inositol
sSPECIAL: semi‐adiabatic spin‐echo full‐intensity acquired localization
Ser: serine
SNR: signal‐to‐noise ratio
STEAM: stimulated‐echo acquisition mode
Tau: taurine
TE: echo time

## 1 INTRODUCTION

γ‐aminobutyric acid (GABA) is the primary inhibitory neurotransmitter in the central nervous system and it is involved in the pathophysiology of many psychiatric and neurological diseases.^1^, ^2^, ^3^ Direct GABA detection by proton magnetic resonance spectroscopy (^1^H‐MRS) is challenging due to its low concentration, complex spin system, and signal overlap with more abundant metabolites such as creatine (Cr), N‐acetylaspartic acid (NAA), glutamate (Glu), and macromolecules (MMs).

With the increase of spectral dispersion and signal‐to‐noise ratio (SNR) at 7 T, short echo‐time (TE) methods using a semiadiabatic spin‐echo full‐intensity acquired localization (sSPECIAL),^4^, ^5^ stimulated echo acquisition mode (STEAM),^6^, ^7^, ^8^, ^9^ or a semilocalized by adiabatic selective refocusing (sLASER) sequence^10^, ^11^ attain high sensitivity by minimizing signal loss due to T_2_ relaxation and J‐evolution, which allows quantification of a large number of metabolites including GABA. Furthermore, many spectral editing methods, such as double quantum filtering,^12^ TE optimization,^13^, ^14^, ^15^ and J‐difference editing^8^, ^16^, ^17^, ^18^ have been proposed for a resolved GABA measurement.

The TE optimization approach uses a point‐resolved spectroscopy sequence (PRESS) with an extended TE (~100 ms) to measure coupled metabolites including GABA over the range of 2.2–2.6 ppm.^13^ In that study, the GABA peak at 2.28 ppm was resolved due to an absence of adjacent Glu multiplets and reduced MM signals. The method displays lower Cramér–Rao lower bound (CRLB) values compared with those obtained by STEAM at short (14 ms) and extended TEs (74 ms). The main limitations of the method are major signal loss because of T_2_ relaxation and chemical shift displacement error of PRESS at ultrahigh magnetic field (≥ 7 T).^14^

Of all the editing methods, the Mescher–Garwood (MEGA) J‐difference editing method is the most commonly used.^19^, ^20^, ^21^ The MEGA‐editing method takes advantage of the J‐couplings between GABA CH_2_ groups at 1.9 and 3.0 ppm. Briefly, selective refocusing editing pulses are applied at 1.9 ppm on alternate scans to refocus side lobes of the GABA triplets at 3.0 ppm, while the Cr singlet is not affected by the editing pulses, resulting in a difference spectrum with eliminated Cr and a resolved GABA resonance at 3.0 ppm. However, due to limited spectral dispersion, these editing pulses unavoidably affect the MM resonances at 1.7 ppm, thus leading to coediting of their coupling partners at 3.0 ppm that overlap with the GABA resonance. Frequency‐selective editing pulses can be applied symmetrically around 1.7 ppm (i.e., at 1.9 and 1.5 ppm on alternate scans) to suppress this coedited MM resonance so that the MM signal at 3.0 ppm is partially affected in the same way and canceled out in the difference spectra.^22^ Because this method relies heavily on the symmetry of the editing frequency, the efficiency of MM subtraction is susceptible to frequency drift. As MMs may vary due to brain tissue type, age, and diseases,^23^ this coedited contamination can be a potential confounding factor for clinical studies in brain pathology.

There are two different approaches to solve these frequency drift and MM contamination issues. The direct use of a highly selective editing pulse is an alternative method if a long pulse duration is allowed in the host localization sequence. A SPECIAL sequence enables longer interpulse delays within the same TE in comparison with a LASER or a sLASER sequence, therefore it has the potential to incorporate long editing pulses. A MEGA‐SPECIAL sequence has been introduced at 3 T with a 30‐ms editing pulse, and it substantially reduced the coedited MM contamination, whereas coedited MM residuals are still inevitable.^24^ GABA editing efficiency is more sensitive to frequency drift with such a narrow band editing pulse.

Second, an asymmetric adiabatic pulse was used to improve the GABA editing efficiency without MM subtraction.^17^ The asymmetric adiabatic pulse provides a broad inversion band, which makes the GABA editing insensitive to both B_1_ and B_0_ variations. A preceding inversion‐recovery pulse was used to suppress MMs at 3.0 ppm. However, the limitations of this method are the requirement for accurate T_1_ knowledge of MM for efficient MM nulling, and further GABA signal loss (~ 50%) due to the partial signal recovery.^6^

Taken together, many efforts have been spent on improving GABA measurements. At 3 T and lower magnetic field, spectral editing methods are generally considered to be the method of choice with which to measure GABA.^25^, ^26^ However, at 7 T, previous short‐TE and editing method comparison studies have shown contradictory results in test–retest assessment.^6^, ^11^ In addition, Dobberthein et al. reported that GABA measurements using short‐TE PRESS with LCModel quantification can be imprecise at 9.4 T due to overlapping peaks,^15^ which suggests that spectral overlaps may still be a limiting factor for short‐TE GABA measurements, even at 7 T. Concerning short‐TE or spectral editing methods, the question remains which method can provide more reliable GABA measurements at 7 T.

Therefore, the purpose of the current study is to compare GABA measurement reproducibility of short‐TE and editing methods in two different brain regions. The sSPECIAL sequence was chosen for the short‐TE spectra acquisition method and the host localization sequence for the MEGA‐editing scheme, because a pair of broadband refocusing pulses in the sSPECIAL sequence mitigates chemical shift displacement error rather than the SPECIAL sequence,^4^, ^27^ and it allows the use of shorter TEs and the incorporation of longer and more selective editing pulses in the same TE than in the sLASER sequence.^28^ In our study, the MEGA‐sSPECIAL scheme was verified using simulation and phantom experiments for further comparison with short‐TE sSPECIAL. In vivo short‐TE sSPECIAL and MEGA‐sSPECIAL sequences were performed twice on the same healthy volunteers in the two brain regions to evaluate the reproducibility of the two methods.

## 2 MATERIALS AND METHODS

### 2.1 Pulse sequence and editing schemes

**FIGURE 1:**
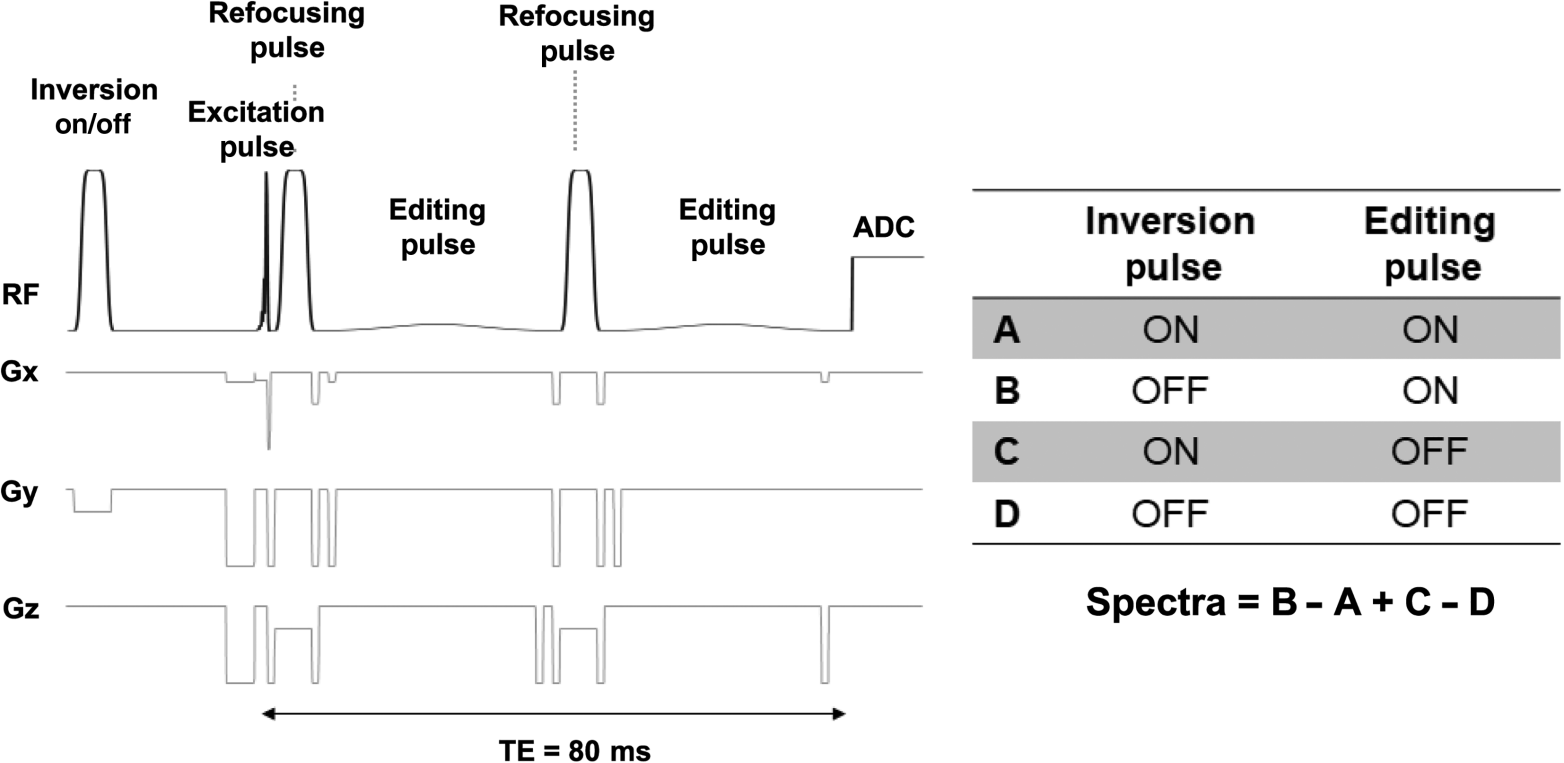
The MEGA‐sSPECIAL pulse sequence diagram (left) and its add‐subtraction scheme (right). Depending on the editing pulse scheme, three editing RF pulses, namely, an asymmetric adiabatic pulse, highly selective Gaussian‐sinc pulse, and Gaussian pulse, are used accordingly

**FIGURE 2:**
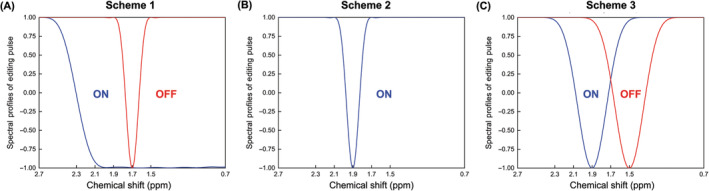
Three macromolecule‐suppressed GABA editing schemes: (A) Scheme 1, edit‐on: an asymmetric adiabatic pulse (at 1.7 ppm), edit‐off: a highly selective Gaussian‐sinc pulse (at 1.7 ppm); (B) Scheme 2, edit‐on: a highly selective Gaussian‐sinc pulse (at 1.9 ppm), edit‐off: no editing pulse; (C) Scheme 3, edit‐on: a Gaussian pulse (at 1.9 ppm), edit‐off: the same Gaussian pulse (at 1.5 ppm)

MEGA‐type editing schemes were implemented in the sSPECIAL localization.^4^ The sequence diagram and add‐subtraction scheme of subspectra are shown in Figure 1. To suppress the coedited MM resonances, three schemes with different editing pulses were implemented and compared (Figure 2). These editing pulses include a 20‐ms asymmetric adiabatic inversion pulse (synthesized using the first half of a 32‐ms hyperbolic secant [HS1] pulse and the second half of an 8‐ms HS4 pulse) with 500 Hz of inversion bandwidth (−1 < M_z_/M_0_ < −0.95) and 136 Hz of transition bandwidth (− 0.95 < M_z_/M_0_ < 0.95) with 1 kHz of peak γB_1_/2π, a highly selective 28‐ms Gaussian pulse with 98 Hz of inversion bandwidth (−1 < M_z_/M_0_ < −0.95) with 35 Hz of peak γB_1_/2π, and a 15‐ms Gaussian pulse (full width half maximum [FWHM] = 120 Hz; γB_1_/2π = 70 Hz). In Scheme 1 (Figure 2A), the asymmetric adiabatic pulse for GABA editing at 1.9 ppm (edit‐on) and highly selective Gaussian pulse for MM subtraction at 1.7 ppm (edit‐off) were used. In Scheme 2 (Figure 2B), as proposed in the previous study,^24^ the highly selective Gaussian pulse was applied at 1.9 ppm for GABA editing (edit‐on) and no editing pulse was applied during edit‐off; MMs around 1.7 ppm were not coedited due to the narrow bandwidth of the editing pulse. In Scheme 3 (Figure 2C), two identical 15‐ms Gaussian pulses were applied symmetrically around 1.7 ppm (at 1.9 and 1.5 ppm in two edit‐on/off alternate scans).^22^ The TE was extended to 80 ms to incorporate a highly frequency‐selective 28‐ms editing pulse. The GABA editing efficiency changes depending on TE were simulated to investigate the effect of extended TE in the MEGA‐sSPECIAL sequence. A maximum 20‐ms long editing pulse can be incorporated in MEGA‐sSPECIAL localization with a TE of 70 ms.

### 2.2 Simulations

Lysine (Lys) was used as a simplified substitute of coedited MMs because Lys is the main contributor to the J‐coupled MM signals at 3.02 and 1.71 ppm in the brain.^29^ Spectral simulations were performed using density matrix formalism implemented in the FID‐A toolkit,^30^ which incorporated RF pulse profiles for localization and editing as well. The chemical shifts and coupling constants of GABA and Lys spin systems were taken from published reports^31^, ^32^ and a linewidth of 2 Hz was used. The frequency offset was varied over a range of ± 60 Hz from the on‐resonance frequency of the respective editing pulse to determine how sensitive the editing efficiency of GABA and coedited MM signals were to the frequency drift.

### 2.3 Phantom experiments

GABA editing and MM subtraction efficiency were validated further by phantom experiments. All the chemicals used in phantoms were purchased from Sigma‐Aldrich (Buchs, Switzerland). Two phantoms, containing 10 mM of GABA or Lys, were prepared respectively. 3‐trimethylsilyl‐propionic acid was added to each phantom as an internal frequency reference and the pH of the solution was adjusted to 7.4. The phantoms were taken out of the refrigerator in advance to set their temperature to room temperature.

All MR measurements were performed on a 7‐T/68‐cm MR scanner (Magnetom, Siemens Medical Solutions, Erlangen, Germany) with a single‐channel quadrature transmitter and a 32‐channel receiver coil (Nova Medical Inc., MA, USA). B_0_ shimming was performed using the first‐ and second‐order shims with FAST (EST)MAP.^33^, ^34^ The gradient echo sequence was used for voxel localization (TR/TE = 6.5/2.82 ms; flip angle = 4; slice thickness = 1 mm; field of view = 25 × 25 cm^2^; matrix size = 256 × 256). The MEGA‐sSPECIAL sequence was applied (with the parameters TR/TE = 15,000/80 ms, average = 24, number of datapoints = 2048, spectral width = 4000 Hz) to compare the three different editing schemes. The host sSPECIAL localization was implemented with a 1.6 ms of asymmetric amplitude‐modulated pulse (P10) for excitation (FWHM = 4 kHz, γB_1_/2π = 1 kHz) and 5.12 ms of HS4‐type adiabatic pulse for refocusing and preinversion (FWHM = 5.1 kHz, γB_1_/2π = 1 kHz). The center of the transmitting frequency was applied at 2.4 ppm to fully cover the spectral width. The transmit voltage was calibrated prior to spectra acquisition to reach the correct flip angle. The outer volume suppression and water suppression were interleaved prior to the MEGA‐sSPECIAL sequence.^35^ The transmit frequency of the respective editing pulse was varied in a range of ± 60 Hz around the resonance frequency of GABA and Lys at 1.9 and 1.7 ppm to investigate the sensitivity of each editing scheme to frequency drift.

### 2.4 In vivo experiments

In vivo measurements were performed in six healthy volunteers (age: 21–33 years; four males and two females) who provided written informed consent under the approval of the Swiss cantonal ethics committee. Each subject was scanned twice to evaluate the test–retest reproducibility of sSPECIAL and MEGA‐sSPECIAL and there was a break of approximately 10 min between the two sessions. The anatomical images were acquired using MP2RAGE^36^ (TR/TE = 6000/4.94 ms, TI1/TI2 = 800/2700 ms, slice thickness = 0.6 mm, field of view = 192 × 192 mm^2^, and matrix size = 256 × 256) to position the MRS voxel. The transmit voltage was calibrated prior to spectra acquisition to reach the correct flip angle. Localized single‐voxel spectra from the motor cortex (M1) and medial prefrontal cortex (mPFC) were acquired by the sSPECIAL (TR/TE = 4000/16 ms) and MEGA‐sSPECIAL (TR/TE = 4000/80 ms) sequences using Scheme 1 (with the parameters average = 128, number of datapoints = 2048, spectral width = 4000 Hz, and volume of interest = 30 × 20 × 20 and 20 × 20 × 25 mm^3^ for the M1 and mPFC, respectively) with four preparation scans. A dielectric pad was positioned on the forehead and the side, respectively, near the mPFC and M1 to improve the transmit field efficiency.^37^ The scanning duration was 8 min 32 s for both sequences. The order of the two sequences was switched in each session.

### 2.5 Data analysis

The raw data were processed using the FID‐A toolkit pipeline.^30^ The receiver channel combination was performed based on the water‐unsuppressed data, weighting the individual channels with the ratio of the signal to the square of the noise.^38^ Eddy current correction was applied. Each measurement block contained four subspectra, as shown in Figure 2. Therefore, first, inversion‐on and ‐off subspectra of each edit‐on/‐off spectra were aligned, then the alignment of edit‐on and ‐off spectra followed. All measurement blocks were then aligned to each other using frequency‐and‐phase correction in the frequency domain using spectral registration to remove the residual Cr peak in the difference spectra, which is necessary for GABA quantification.^30^, ^39^

3‐Hz exponential line broadening was applied for phantom spectra. The signal intensities of GABA and Lys were calculated by the signal integral in the 2.85 to 3.15 ppm range and scaled using min‐max normalization. No zero filling or apodization was applied to in vivo data. All spectra were fitted using LCModel with simulated basis sets. The unsuppressed water signal was used as an internal reference for absolute quantification. For the sSPECIAL spectra, an experimentally measured MM spectrum^40^ and simulated spectra of the following 21 metabolites were included in the basis set for LCModel analysis: alanine (Ala), ascorbate (Asc), aspartate (Asp), Cr, GABA, glucose (Glc), glutamine (Gln), Glu, glycerophosphorylcholine (GPC), glycine (Gly), glutathione (GSH), lactate (Lac), myo‐inositol (mIns), NAA, N‐acetylaspartylglutamate (NAAG), phosphocreatine (PCr), phosphorylcholine (PCho), phosphorylethanolamine (PE), scyllo‐inositol (sIns), serine (Ser), and taurine (Tau). The MEGA edit‐off spectra were fitted using the simulated basis set at a TE of 80 ms with the same number of metabolites as the sSPECIAL spectra without the MM spectrum. The difference spectra were fitted with a simulated basis set of five metabolites, including GABA, Glu, Gln, NAA, and NAAG. The spectral range for LCModel analysis was set from 1.8 to 4.2 ppm. All metabolite levels were reported in institutional units (IUs). Tissue segmentation was performed from the reference MP2RAGE image using SPM12^41^ for partial volume correction. T_2_ relaxation times of all metabolites from the literature are listed in the supporting information (ST1). T_2_ relaxation correction was applied for each metabolite concentration, including tissue correction.^42^ The SNR was calculated using the height of NAA peak at 2.01 ppm divided by the standard deviation (SD) of the noise between 9.5 and 10.0 ppm; 32, 64, and 96 spectra were selected arbitrarily from the original 128 spectra and averaged to assess changes in reproducibility depending on the number of averaged spectra across the scans. The tCr (total creatine) signal in the edit‐off (MEGA‐off) spectrum was used as an internal reference for the GABA/tCr ratio of difference (MEGA‐diff) spectra.

### 2.6 Statistical analysis

All statistical analyses were performed using Python 3. The mean coefficient of variation (CV) was used to evaluate between‐session reproducibility. Pearson's correlation coefficients (*r*) between pairs of measurements (the first and second scans) and between methods were calculated within each brain region (the M1 and mPFC) and across both brain regions accompanied by significance tests.^43^ The intraclass correlation coefficient (ICC) was used to access the consistency of between‐session measurements using a two‐way mixed model.^44^ A Bland–Altman analysis was applied to evaluate the agreement of the two measurement methods (sSPECIAL and MEGA‐sSPECIAL).^45^

## 3 RESULTS

**FIGURE 3:**
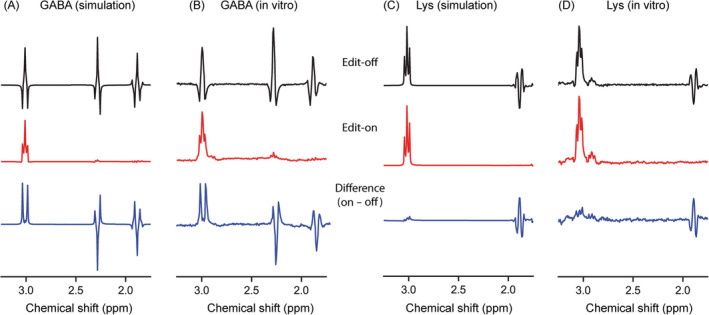
Simulated and in vitro γ‐aminobutyric acid (GABA) and lysine (Lys) MR spectra obtained using MEGA‐sSPECIAL with asymmetric adiabatic and narrow Gaussian‐sinc pulses (Scheme 1)

Simulated GABA and Lys spectra were in good agreement with the spectra obtained in vitro (Figure 3A,B). The differences of normalized GABA signals between the simulation and in vitro results were 0.03 ± 0.04 (Scheme 1), 0.02 ± 0.02 (Scheme 2), and 0.03 ± 0.03 (Scheme 3). For Lys, the differences were −0.04 ± 0.08 (Scheme 1), −0.03 ± 0.04 (Scheme 2), and −0.04 ± 0.10 (Scheme 3). The difference spectra demonstrate the subtraction of Lys peak at 3.0 ppm. The chemical shift and J‐coupling constants of Lys used in the simulation were obtained from Deelchand et al.,^32^ and did not include the information of the small resonance peak at 2.9 ppm. This peak was coedited when the inversion pulse was applied at 1.7 ppm and was cosuppressed in the difference spectra as well.

**FIGURE 4:**
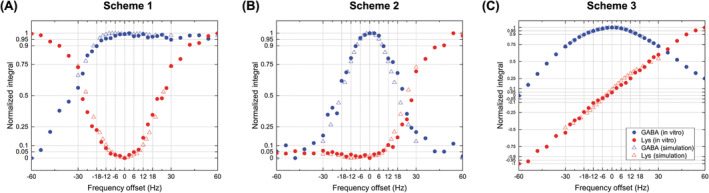
Normalized signal integrals in the range of 2.85–3.15 ppm for GABA (blue) and Lys (red) signals obtained by simulations (open triangles) and in vitro experiments (filled circles) to investigate the editing efficiency of (A–C) three macromolecule‐suppressed GABA‐editing schemes to B_0_ frequency drift by varying the frequency offset by ± 60 Hz around the on‐resonance frequency of each editing pulse

The sensitivity of GABA and Lys editing efficiency to frequency drifts was evaluated using simulations and phantom experiments for three different schemes (Figure 4). The water linewidth (FWHM) was 3.5 Hz, showing good multiplet resolution in in vitro spectra. The GABA signal loss was less than 5% over the frequency range from −15 to +60 Hz for Scheme 1, from −6 to +6 Hz for Scheme 2, and from −8 to +8 Hz for Scheme 3, respectively. The Lys signal increased by within 5% over the frequency range from −8 to +8 Hz for Scheme 1, from −60 to +6 Hz for Scheme 2, and from −2 to +2 Hz for Scheme 3, respectively. The editing efficiencies of each scheme were: Scheme 1 = 0.5; Scheme 2 = 0.45; and Scheme 3 = 0.5. The MEGA‐sSPECIAL sequence was simulated for several TEs (68, 70, 74, 80, 90, and 100 ms). The GABA editing efficiency changed from 0.44 to 0.50 with TEs and achieved its maximum at a TE of 80 ms. By extending TE from 68 to 80 ms, the GABA signal was reduced by 5.8% due to T_2_ relaxation. However, the GABA editing efficiency at a TE of 80 ms increased by 3.0% relative to that at 68 ms, which partially compensated for the signal loss due to T_2_ relaxation.

The transmitting frequency drift is dependent on the system stability and the duty cycle of the gradient, which can increase by up to 2 Hz/min after functional magnetic resonance imaging studies.^46^, ^47^ Considering both GABA editing and MM subtraction efficiencies, Scheme 1 with the asymmetric adiabatic and narrow Gaussian pulse combination was the least sensitive method to frequency drift, showing a frequency range of ± 8 Hz within the given 5% threshold. Based on the simulation and in vitro validation of the editing schemes, Scheme 1 was used for further reproducibility measurement in vivo for comparisons with the sSPECIAL sequence at short TE.

The average water linewidth and SNR for both measurement methods in the two brain regions are presented in Table 1. There was no significant difference between the sessions. The fractional tissue compositions in the M1 were 0.30/0.68/0.02 (gray matter [GM]/white matter [WM]/cerebrospinal fluid [CSF]) for the first session and 0.30/0.69/0.01 (GM/WM/CSF) for the second session. The fractional tissue compositions in the mPFC were 0.55/0.21/0.27 (GM/WM/CSF) for the first session and 0.56/0.20/0.24 (GM/WM/CSF) for the second session. There was no significant difference in tissue composition between the two sessions. The MRS voxel placement and rotational angle are reported in the supporting information (ST2).

**TABLE 1:** Mean water linewidth and signal‐to‐noise ratio (SNR) for each measurement. There was no significant difference between sessions

		M1		mPFC	
		1st	2nd	1st	2nd
**Linewidth (Hz)**	sSPECIAL	12.0 ± 0.8	11.9 ± 0.4	12.6 ± 1.1	13.7 ± 2.1
	MEGA‐sSPECIAL	12.4 ± 0.9	12.1 ± 1.0	12.7 ± 0.7	12.5 ± 1.2
**SNR**	sSPECIAL	548 ± 51	587 ± 86	486 ± 59	504 ± 32
	MEGA‐off	235 ± 60	236 ± 53	257 ± 34	272 ± 28
	MEGA‐diff	171 ± 35	176 ± 45	163 ± 22	183 ± 22

**FIGURE 5:**
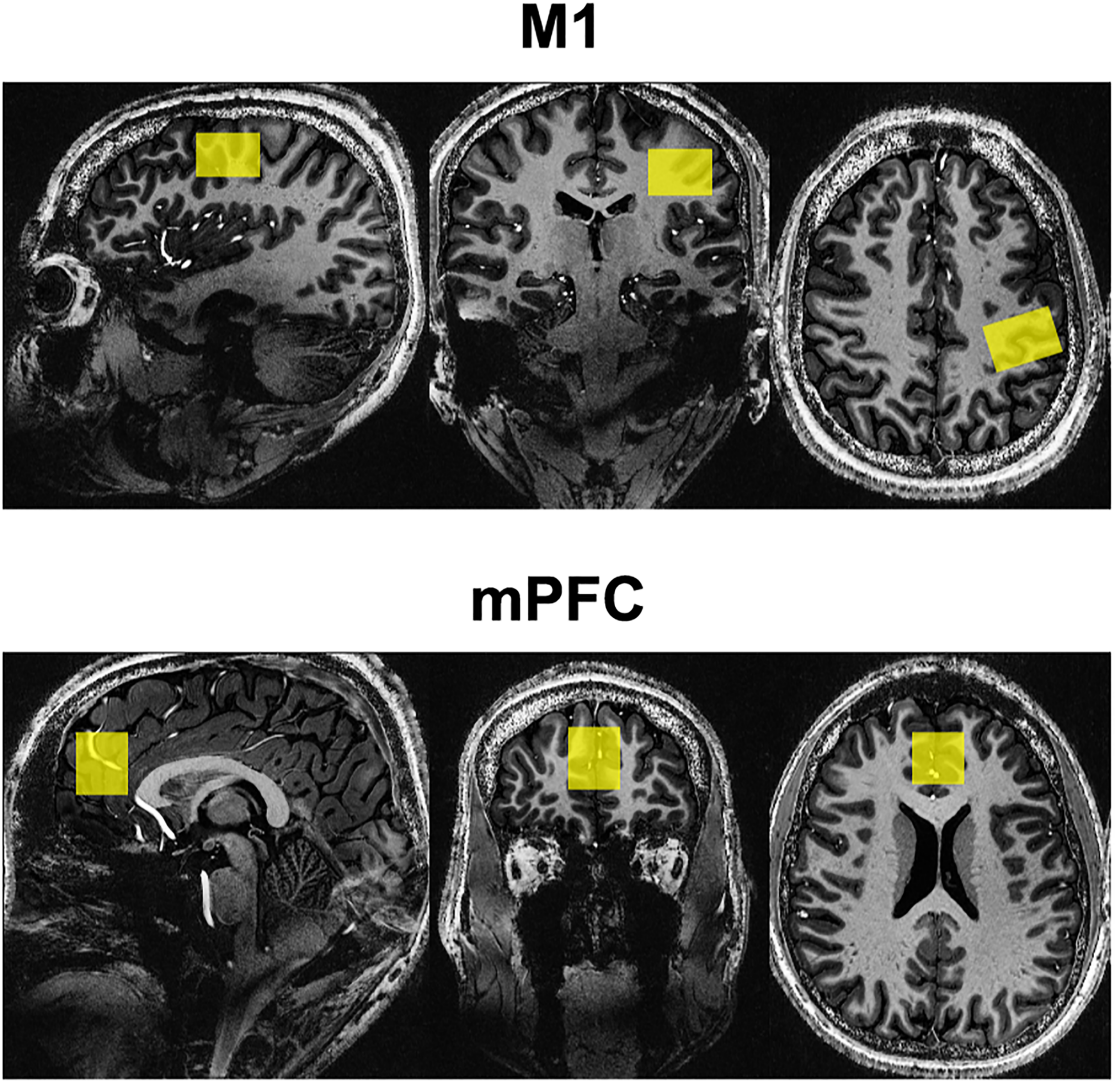
The MRS voxel placements are superimposed on the three planes for the motor cortex (M1) (30 x 20 x 20 mm^3^) (top) and medial prefrontal cortex (mPFC) (20 x 20 x 25 mm^3^) (bottom)

**FIGURE 6:**
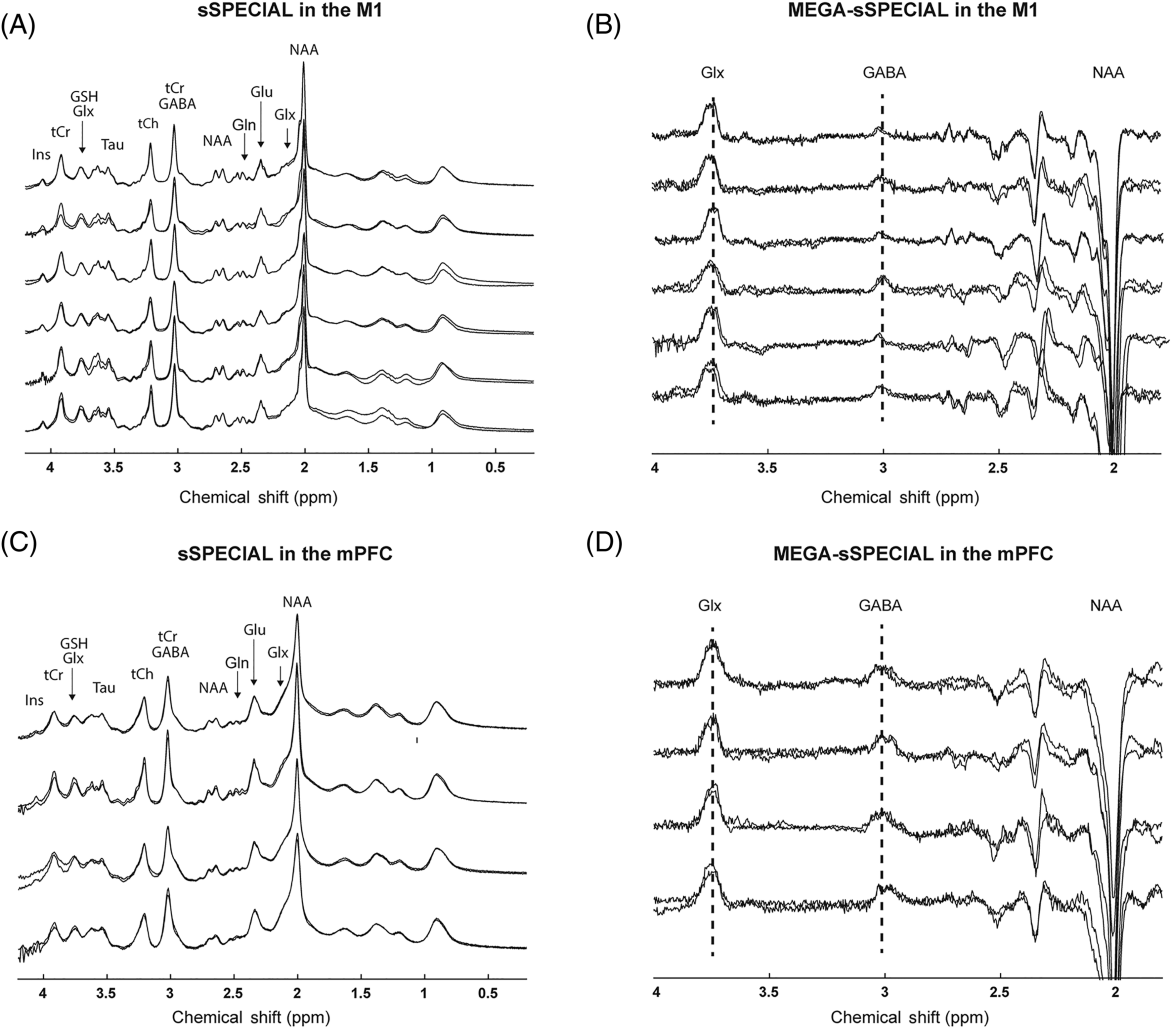
Individual spectra acquired using the (A and C) sSPECIAL and (B and D) MEGA‐sSPECIAL sequences in both brain regions. The spectra from the first and second sessions are overlaid for each volunteer. GABA, γ‐aminobutyric acid; Gln, glutamine; Glu, glutamate; Glx, Glu+Gln; GSH, glutathione; Ins, myo‐Inositol; NAA, N‐acetylaspartic acid;Tau, taurine; tCh, total choline; tCr, total creatine

The representative MR images with voxel placements are presented in yellow for both regions in Figure 5. The individual spectra acquired using sSPECIAL and MEGA‐sSPECIAL in the M1 and mPFC are illustrated in Figure 6A–D. Each spectrum corresponds to individual subjects, and spectra in the first and second sessions are overlaid in the figure. Due to motion artifacts and lipid contamination, two spectra acquired in the mPFC are excluded from the results. The absence of Cho peak at 3.2 ppm in the difference spectra indicated subtraction of residual Cr signal at 3.03 ppm.^48^ The mean concentration of each metabolite with SD and mean CV are reported in Tables 2 and 3. The GABA levels were 1.56 ± 0.49 and 1.01 ± 0.09 in the mPFC and 0.92 ± 0.26 and 0.51 ± 0.05 in the M1 by sSPECIAL and MEGA‐sSPECIAL, respectively. A GABA concentration comparison with other values from the literature is provided in Table 4. All the editing methods reported in the table use the MM suppression scheme.

**TABLE 2:** Mean concentrations (institutional units [IUs]), SD, mean Cramér–Rao lower bound (CRLB), and mean test–retest coefficients of variation (CVs) of metabolites with CRLB < 25% in the motor cortex (M1) region

Method	Metabolite	1st			2nd			Mean CV (%)
		Mean conc. (IUs)	SD	Mean CRLB (%)	Mean conc. (IUs)	SD	Mean CRLB (%)	
**sSPECIAL**	**Asp**	1.34	0.53	17.7	1.52	0.25	15.8	17.4
	**Lac**	0.75	0.32	7.8	0.81	0.07	8.0	7.1
	**GABA**	0.94	0.41	18.7	0.91	0.21	15.7	21.8
	**mIns**	5.86	0.71	2.5	5.35	0.55	2.2	7.9
	**PE**	1.81	0.20	6.0	2.02	0.35	5.9	7.3
	**Gly**	0.34	0.18	19.5	0.37	0.18	19.2	15.3
	**Tau**	1.08	0.25	12.8	1.09	0.12	8.8	11.0
	**NAA**	11.28	0.61	1.3	11.09	0.52	1.0	1.5
	**NAAG**	2.53	0.46	4.0	2.34	0.21	4.8	7.5
	**Gln**	1.05	0.52	15.7	1.13	0.42	11.0	12.7
	**Glu**	8.80	1.27	2.2	8.96	0.90	1.7	6.5
	**GSH**	1.09	0.08	4.8	1.01	0.17	5.5	8.8
	**tNAA**	13.81	0.65	1.2	13.41	0.57	1.0	2.0
	**Glx**	9.85	1.44	2.5	10.09	0.94	2.0	5.4
	**tCho**	1.36	0.13	2.5	1.24	0.18	2.2	9.2
	**tCr**	7.37	0.22	1.3	7.01	0.17	1.2	3.5
	**GABA/tCr**	0.13	0.05	‐	0.13	0.03	‐	21.6
**MEGA‐off**	**Asp**	0.72	0.18	23.0	0.84	0.21	19.7	20.9
	**NAA**	11.07	1.05	1.3	10.70	0.44	1.2	7.1
	**NAAG**	3.07	0.43	4.5	2.99	0.46	5.7	5.3
	**Gln**	1.18	0.23	22.8	1.38	0.26	18.2	22.4
	**Glu**	8.75	1.05	3.2	8.64	1.00	3.8	7.6
	**GSH**	0.65	0.09	12.8	0.60	0.11	16.7	8.6
	**tNAA**	14.14	1.38	1.0	13.69	0.54	1.0	4.3
	**tCho**	1.45	0.19	2.2	1.30	0.22	2.2	9.7
	**Glx**	9.75	1.28	4.3	10.02	1.08	4.3	9.9
	**tCr**	7.01	0.59	1.2	6.80	0.44	1.2	4.3
**MEGA‐diff**	**GABA**	0.53	0.04	9.3	0.50	0.04	11.7	4.1
	**GABA/tCr**	0.08	0.01	‐	0.07	0.00	‐	5.8

Abbreviations: Asp, aspartate; GABA, γ‐aminobutyric acid; Gln, glutamine; Glu, glutamate; Glx, Glu + Gln; Gly, glycine; GSH, glutathione; Lac, lactate; mIns, myo‐inositol; NAA, N‐acetylaspartic acid; NAAG, N‐acetylaspartylglutamate; PE, phosphorylethanolamine; Tau, taurine; tCho, total choline; tCr, total creatine (PCr + Cr); tNAA, total NAA (NAA + NAAG).

**TABLE 3:** Mean concentrations with SD of metabolites detected with Cramér–Rao lower bound (CRLB) < 25% in the medial prefrontal cortex (mPFC) region. The mean test–retest coefficients of variation (CVs) are also listed

Method	Metabolite	1st			2nd			Mean CV (%)
		Mean conc. (IU)	SD	Mean CRLB (%)	Mean conc. (IU)	SD	Mean CRLB (%)	
**sSPECIAL**	**Asp**	1.45	0.29	13.5	1.56	0.31	17.0	5.1
	**Lac**	0.69	0.10	13.3	0.75	0.12	7.5	12.0
	**GABA**	1.46	0.43	13.0	1.56	0.52	9.8	10.2
	**mIns**	3.89	0.47	4.0	4.20	0.37	2.3	5.6
	**PE**	2.68	0.31	5.8	2.36	0.55	4.8	10.4
	**Gly**	0.59	0.25	20.0	0.48	0.04	15.5	22.7
	**Tau**	1.13	0.30	13.3	1.14	0.32	9.4	1.7
	**NAA**	10.16	0.54	1.0	10.45	0.71	1.0	3.5
	**NAAG**	0.72	0.02	11.0	0.84	0.09	7.3	10.0
	**Gln**	1.61	0.19	8.5	1.50	0.15	7.5	8.7
	**Glu**	9.64	0.72	2.0	9.03	0.91	1.8	6.9
	**GSH**	0.91	0.18	7.0	0.80	0.10	6.3	10.2
	**tNAA**	10.88	0.51	1.0	11.29	0.63	1.0	3.2
	**Glx**	11.34	0.59	2.0	10.98	1.42	2.0	5.9
	**tCho**	0.70	0.19	4.0	0.75	0.11	2.8	8.3
	**tCr**	6.60	0.85	2.0	6.67	0.95	1.5	4.4
	**GABA/tCr**	0.24	0.08	‐	0.26	0.11	‐	11.2
**MEGA‐off**	**Asp**	1.33	0.11	20.3	1.33	0.19	20.8	6.4
	**NAA**	9.17	0.97	1.3	9.67	0.56	1.3	5.4
	**NAAG**	1.03	0.35	8.3	1.25	0.41	6.5	13.4
	**Gln**	1.20	0.19	22.5	1.20	0.36	25.5	13.2
	**Glu**	9.21	1.50	4.0	9.56	1.24	3.5	9.3
	**GSH**	0.51	0.22	19.5	0.56	0.22	20.3	12.8
	**tNAA**	10.04	0.95	1.0	11.15	1.11	1.0	8.8
	**tCho**	0.86	0.22	2.8	0.88	0.12	2.3	9.8
	**Glx**	10.48	1.50	4.5	10.84	1.52	4.3	7.0
	**tCr**	6.33	0.97	1.5	6.73	0.82	1.3	1.4
**MEGA‐diff**	**GABA**	1.03	0.22	8.8	1.05	0.30	9.7	5.8
	**GABA/tCr**	0.15	0.04	‐	0.15	0.05	‐	6.4

**TABLE 4:** Comparisons of GABA concentrations and GABA/tCr ratios with standard deviation. All the editing sequences used a macromolecule (MM) subtraction scheme

Sequence	Position (ml)	TE (ms)	GABA (IUs)	GABA/tCr
sSPECIAL (this study)	mPFC (12)	16	1.56 ± 0.49	0.25 ± 0.9
MEGA‐sSPECIAL (this study)	mPFC (12)	80	1.01 ± 0.09	0.12 ± 0.07
STEAM^8^, ^a^	DLPFC (27)	14	2.08 ± 0.5	‐
STEAM^8^, ^b^	DLPFC (27)	14	2.24 ± 0.6	
sLASER^49^, ^a^	mPFC (15.6)	74	1.6 ± 0.6 mM	‐
sLASER^49^, ^b^	mPFC (15.6)	74	1.3 ± 0.5 mM	‐
sLASER^11^	DLPFC (8)	38	‐	0.22 ± 0.09
MEGA‐sLASER^11^	DLPFC (8)	80	‐	0.10 ± 0.02
STEAM^50^	left DLPCF (7.9)	14	1.72 ± 0.34	‐
sSPECIAL (this study)	MC (10)	16	0.92 ± 0.26	0.13 ± 0.05
MEGA‐sSPECIAL (this study)	MC (10)	80	0.51 ± 0.05	0.08 ± 0.03
sLASER^11^	MC (8)	38	‐	0.16 ± 0.03
MEGA‐sLASER^11^	MC (8)	80	‐	0.11 ± 0.05
MEGA‐sLASER^51^	MC (9)	72	‐	0.11 ± 0.02
LASER^52^	MC (8)	35	0.8 ± 0.6 mM	‐

Abbreviations: DLPFC, dorsolateral prefrontal cortex; IUs, institutional units; LASER, localized by adiabatic selective refocusing; MC, motor cortex.

^a^ First session.

^b^ Second session.

**FIGURE 7:**
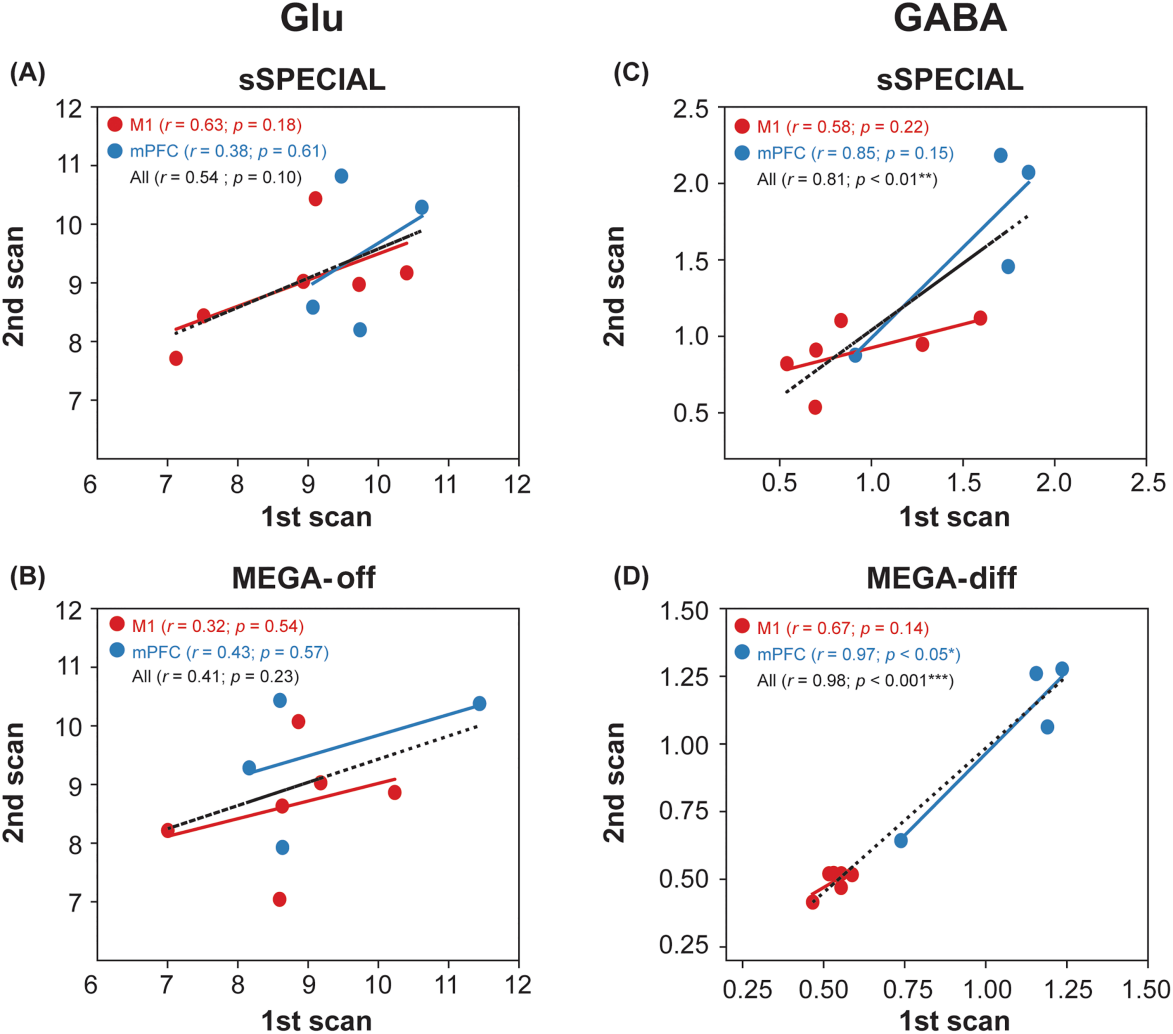
Pearson's correlation coefficients (*r*) and statistical significances are shown for γ‐aminobutyric acid (GABA) and glutamate (Glu) measurements to compare the test–retest reliability between the two sessions (first and second). The data obtained in the motor cortex (M1) and medial prefrontal cortex (mPFC) are marked in red and blue, respectively. *p < 0.05; **p < 0.01; ***p < 0.001

**FIGURE 8:**
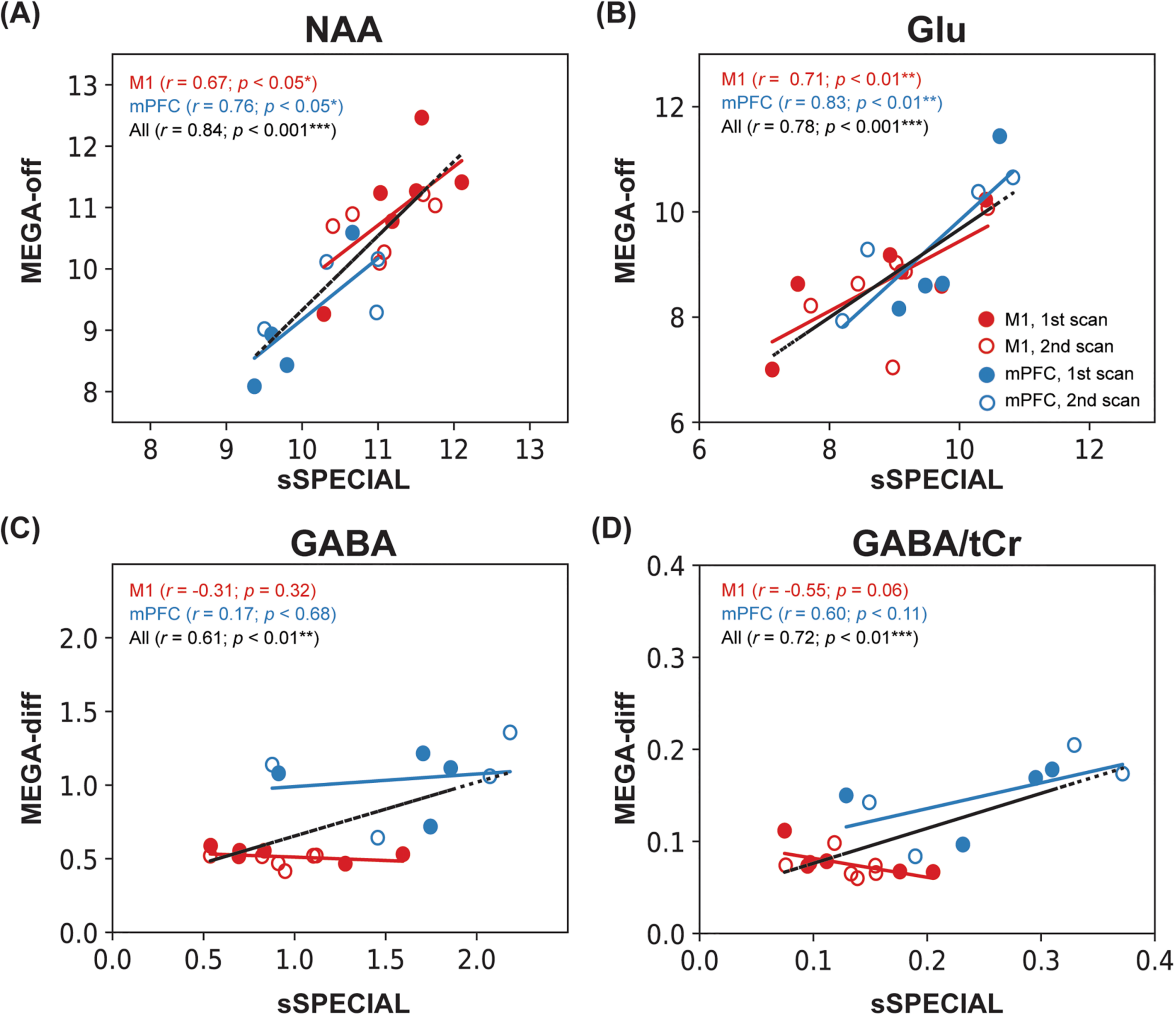
Correlation plots for (A) N‐acetylaspartic acid (NAA): sSPECIAL versus MEGA‐off, (B) for glutamate (Glu): sSPECIAL versus MEGA‐off, (C) for γ‐aminobutyric acid (GABA): sSPECIAL versus MEGA‐diff, and (D) for GABA/tCr: sSPECIAL versus MEGA‐diff are presented. Metabolite concentrations measured in the motor cortex (M1) are displayed as red‐filled (first session) and empty circles (second session), and in the medial prefrontal cortex (mPFC) they are displayed as blue‐filled (first session) and empty circles (second session). Each key shows the *r* value for each region separately, and for both regions pooled together. *p < 0.05; **p < 0.01; ***p < 0.001

**FIGURE 9:**
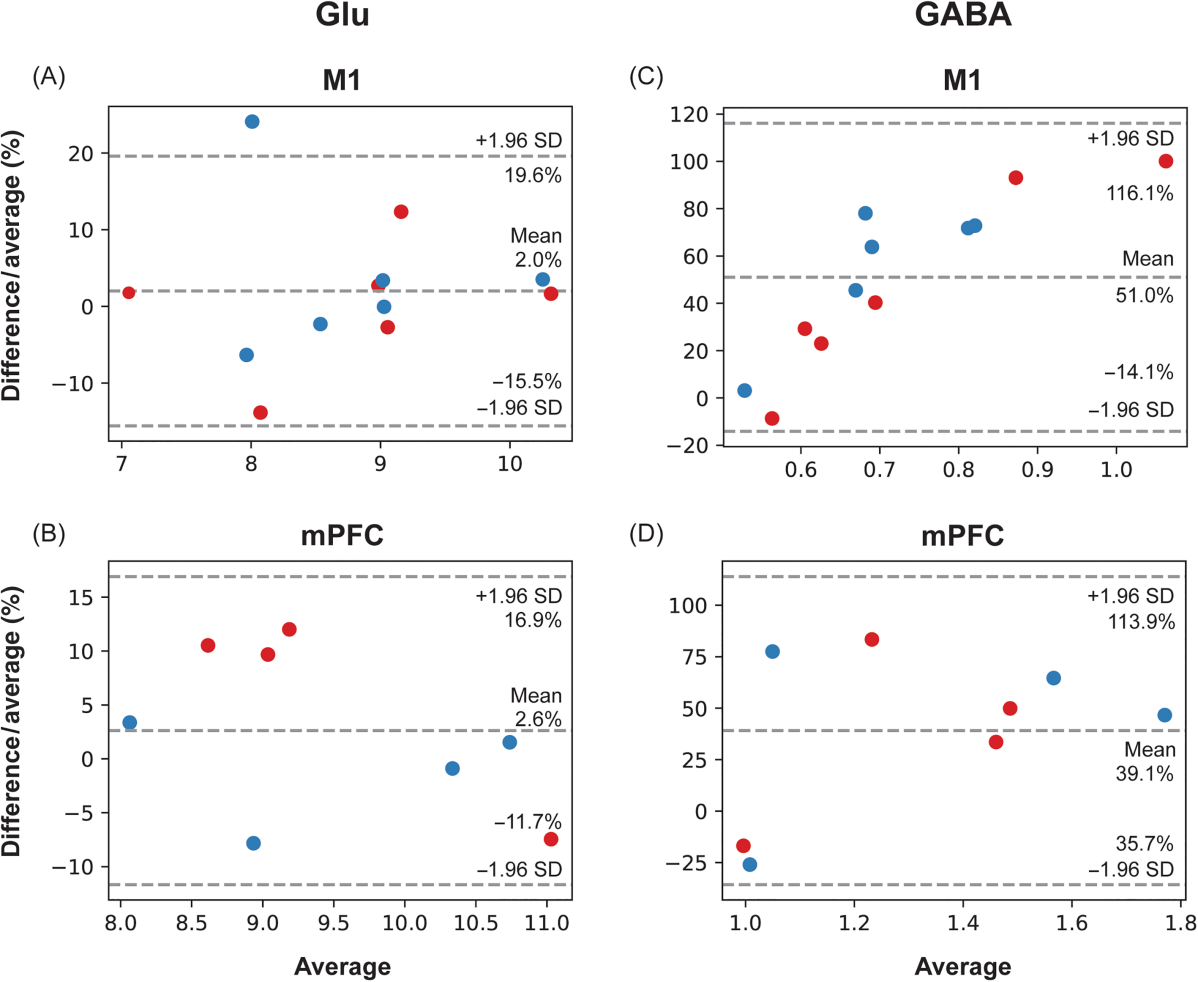
Bland–Altman plots showing agreement between short echo‐time (TE) and MEGA‐sSPECIAL methods within all subjects and both scans regarding (A and B) Glutamate (Glu) and (C and D) γ‐aminobutyric acid (GABA). The first (in red) and second (in blue) session measurements are considered to be independent. Each central dotted line indicates the mean value of the differences of the paired data, and the upper and lower lines show the 95% confidence interval, respectively

The between‐session reliability was evaluated using CV (Tables 2 and 3), Pearson's *r* correlation coefficients (Figure 7), and ICC (Table 5). Pearson's *r* correlation coefficients (Figure 8) and Bland–Altman plots (Figure 9) were used to assess the agreement between the paired datasets acquired using the two different sequences. A Pearson's correlation coefficient of between 0.2 and 0.39 was regarded as weak, between 0.4 and 0.7 as moderate, and above 0.7 as strong correlation.^42^ ICC values of less than 0.4 were considered poor, between 0.4 and 0.6 as moderate, between 0.6 and 0.75 as good, and above 0.75 as excellent.^53^ Pearson's *r* correlation coefficients and ICCs were calculated in the two regions separately and across the regions to increase the dynamic range for correlation assessment.

**TABLE 5:** Between‐session (the first and second measurements) intraclass correlation coefficients (ICCs) for sSPECIAL and MEGA‐sSPECIAL in the motor cortex (M1) and medial prefrontal cortex (mPFC). Glutamate (Glu) and N‐acetylaspartic acid (NAA) concentrations were quantified using MEGA‐off spectra and γ‐aminobutyric acid (GABA) was quantified using MEGA‐diff spectra

	M1		mPFC		All	
	sSPECIAL	MEGA‐sSPECIAL	sSPECIAL	MEGA‐sSPECIAL	sSPECIAL	MEGA‐sSPECIAL
**GABA**	**0.49**	**0.82**	**0.81**	**0.92**	**0.82**	**0.98**
**Glu**	**0.60**	0.32	0.31	**0.42**	**0.54**	**0.41**
**NAA**	**0.92**	0.30	**0.41**	0.31	**0.77**	**0.59**

*Note*: ICC classification: poor (ICC < 0.4), fair (0.4 ≤ ICC < 0.60, good (0.6 ≤ ICC < 0.75), and excellent (0.75 ≤ ICC) reliability. Fair and good correlations are in bold and excellent correlations are in bold and underlined.

Abbreviations: MEGA.

The test–retest reliability of Glu and GABA measurements is demonstrated in Figure 7 and Table 5. Regarding GABA measurements using MEGA‐sSPECIAL, there was a moderate correlation trend in the M1 (*r* = 0.67, *p* = 0.14; ICC = 0.82; CV = 4.1%), and strong correlations in the mPFC (*r* = 0.97, *p* < 0.05; ICC = 0.92; CV = 5.8%) and across both regions (*r* = 0.98, *p* < 0.001; ICC = 0.98). There were moderate and strong correlation trends in the M1 (*r* = 0.58, *p* = 0.22; ICC = 0.49; CV = 21.8%) and in the mPFC (*r* = 0.85, *p* = 0.18; ICC = 0.81; CV = 10.2%), and strong correlation across both regions (*r* = 0.81, *p* < 0.01; ICC = 0.82) with sSPECIAL.

In the case of Glu measurements, the sSPECIAL sequence showed a moderate correlation tendency between sessions in the M1 (*r* = 0.63, *p* = 0.18; ICC = 0.60; CV = 6.5%) and overall regions (*r* = 0.54, *p* = 0.10; ICC = 0.54). The MEGA‐off sequence did not show significant between‐session correlation in the M1 (*r* = 0.32, *p* = 0.54; ICC = 0.32; CV = 7.6%), in the mPFC (*r* = 0.43, *p* = 0.57; ICC = 0.42; CV = 9.3%), or across both regions (*r* = 0.41, *p* = 0.23; ICC = 0.41). Correlation plots to assess intersequence agreement for Glu, NAA, GABA, and GABA/tCr measurements are illustrated in Figure 8. There was a strong correlation between the sSPECIAL and MEGA‐off measurements of Glu in the M1 (*r* = 0.71; *p* < 0.01) and in the mPFC (*r* = 0.83; *p* < 0.01) individually and across both regions (*r* = 0.78; *p* < 0.001). NAA measurements by the two sequences were strongly correlated in the M1 (*r* = 0.67; *p* < 0.05), mPFC (*r* = 0.76; *p* < 0.05), and across both areas (*r* = 0.84; *p* < 0.001). GABA and GABA/tCr measurements were inversely and moderately correlated in the M1 (*r* = −0.31, *p* = 0.32; *r* = −0.55, *p* = 0.06) and overall strongly correlated across the regions (*r* = 0.61, *p* < 0.01; *r* = 0.72, *p* < 0.01). The Bland–Altman plots illustrating the differences of the two measurement methods for Glu and GABA measurements are presented in Figure 9.

**FIGURE 10:**
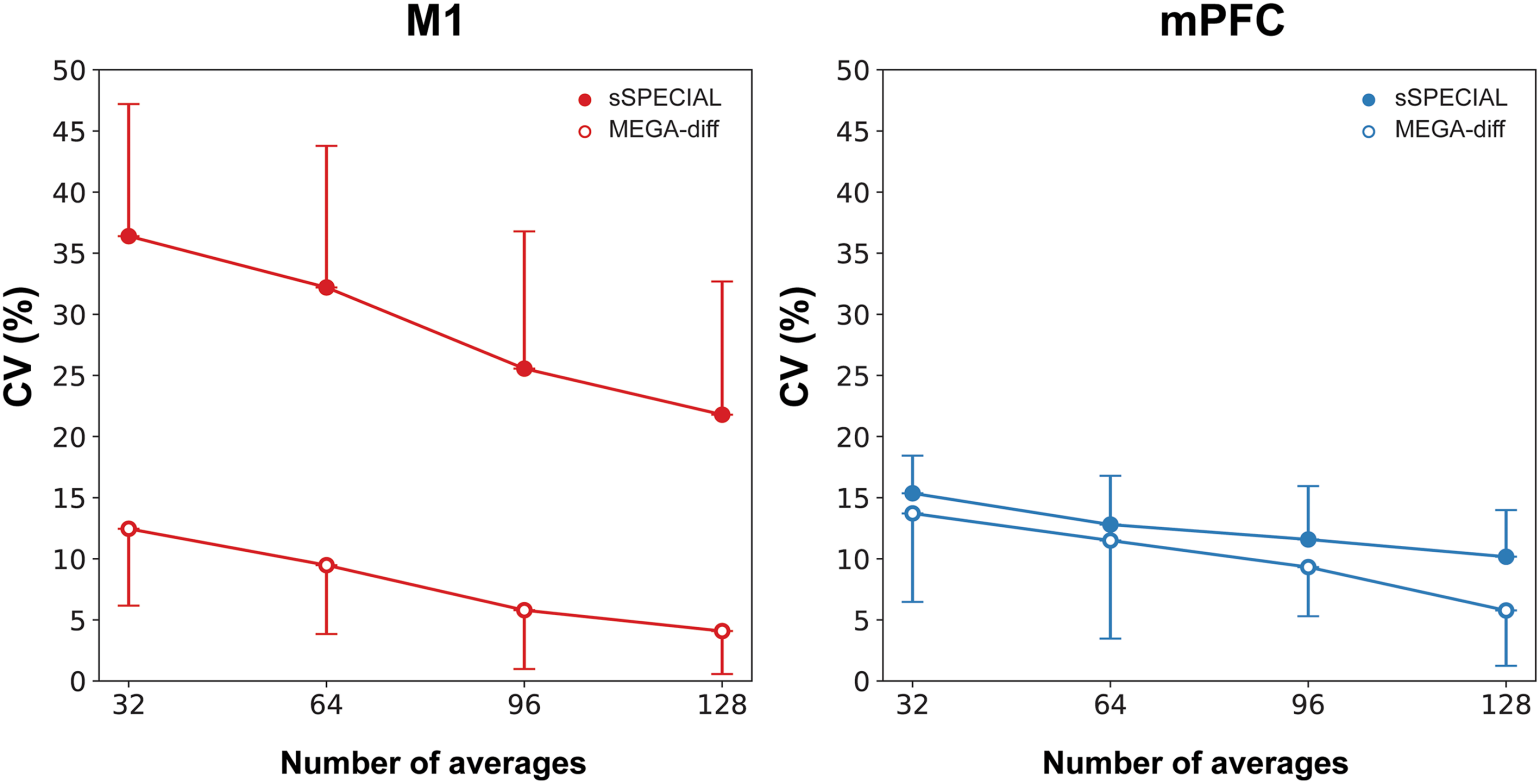
Coefficients of variation (CVs) of GABA were obtained with averages of 32, 64, 96, and 128 in the motor cortex (M1) and medial prefrontal cortex (mPFC). The mean CVs are presented in the figure and the error bars show SD. For visualization purposes, only positive SD values for sSPECIAL and negative SD values for MEGA‐diff are illustrated. CV changes over the number of averaged spectra are evaluated in both regions by the two methods. The measured concentrations of GABA are 60% higher in the mPFC than those in the M1

CV changes depending on the number of averages in the M1 and mPFC using sSPECIAL and MEGA‐sSPECIAL are presented in Figure 10. With the increase in the number of averages from 32 to 128, the CVs of GABA measured in the M1 decreased from 36.4% to 21.8% using sSPECIAL and from 12.5% to 4.1% using MEGA‐sSPECIAL. The CVs of GABA measured in the mPFC decreased from 15.4% to 10.7% using sSPECIAL and from 13.7% to 4.3% using MEGA‐sSPECIAL.

## 4 DISCUSSION

In this study, we introduced a GABA‐resolved measurement by the MEGA‐sSPECIAL method at 7 T with the subtraction of coedited MM signal and evaluated between‐session reproducibility and intersequence agreement between the MEGA‐sSPECIAL and nonediting short‐TE methods in vivo.

### 4.1 In vivo GABA measurements in the M1 and mPFC

At 7 T, even although NAAG, Glu, and Gln could now be detected with low CRLB (< 20%), detecting low concentrated and overlapping metabolite signals such as GABA, Gly, or GSH was still challenging.^54^ The reported mean GABA concentrations at 7 T were 1.6 mM by the sLASER sequence in the mPFC^49^ and 0.8 mM in the M1 by the LASER sequence.^52^ GABA/tCr ratios at 7 T were 0.14 in the mPFC by sLASER^55^ and 0.11–0.16 in the M1 by sLASER and MEGA‐sLASER.^11^ In our study, the GABA levels obtained were 1.56 ± 0.49 and 1.01 ± 0.09 in the mPFC and 0.92 ± 0.26 and 0.51 ± 0.05 in the M1 by sSPECIAL and MEGA‐sSPECIAL, respectively. The mean concentrations of quantified metabolites reported in both brain regions in our study lie within the range of recently published literature.^52^, ^55^, ^56^

Hong et al.^11^ reported higher GABA and GABA/tCr values by sLASER than those of MEGA‐sLASER at 7 T. In this study, MMs were not included in the basis set of sLASER, and a Lorentzian lineshape singlet peak to mimic MM signal at 3 ppm was included in the basis set of MEGA‐sLASER. Even although the measured MM spectrum was included in the basis set of the short‐TE sSPECIAL, the measurement accuracy may still be affected by the estimation of spline baseline (e.g., in LCModel).^57^

CRLBs for GABA were in the range of 16.7%–18.7% using sSPECIAL and 9.4%–11.6% using MEGA‐sSPECIAL in the M1. They were in the range of 9.8%–13.0% using sSPECIAL and 8.8%–9.7% using MEGA‐sSPECIAL in the mPFC. Relative CRLB values have been used to estimate uncertainties and exclude poor quality data. However, especially for low concentrated metabolites with overlapped peaks like GABA, the use of CRLB as a quality control threshold can result in a biased estimation of metabolite concentrations.^58^ It has been shown that even with low percentage CRLB values such as 2%–7%, LCModel concentration estimation can be imprecise by overestimating/underestimating the levels of metabolites in short‐TE measurements.^15^, ^57^

### 4.2 Between‐session reproducibility and intersequence agreement

To our knowledge, there are very few reproducibility studies that have reported GABA levels at 7 T in multiregions using different measurement methods. Prinsen et al. reported a test–retest GABA CV of 9.5% in the occipital cortex using sLASER localization with inversion recovery.^6^ Gu et al. reported a test–retest GABA/tCr CV of 7% using the MEGA‐SPECIAL sequence (Scheme 2) at 3 T for 8 min 40 s of acquisition time.^24^ The mean CVs of GABA measurements by sSPECIAL were 21.5% in the M1 and 10.2% in the mPFC. This is comparable with the CV of 9.5% by the global inversion‐preparation method by Prinsen et al. in the occipital cortex, where the GABA concentration was higher than that of the M1 and similar to the one in the mPFC. On the other hand, the mean CVs of GABA measurements by MEGA‐sSPECIAL were 4.1% in the M1 and 5.8% in the mPFC, which suggests that MEGA‐sSPECIAL (Scheme 1) has better measurement reproducibility compared with sSPECIAL and a previously reported study using Scheme 2.^24^

Furthermore, between‐session Pearson's correlation coefficients showed strong correlation for GABA measurements across regions by both sSPECIAL (*r* = 0.81, *p* < 0.01) and MEGA‐diff (*r* = 0.98, *p* < 0.001), where MEGA‐diff displayed an even better performance. Even although the regional correlation coefficients were not statistically significant, this may be due to the small sample size and limited range of observation within individual regions, which influences the correlation coefficients.^59^ On the other hand, the ICC of MEGA‐diff (0.98) is also better than that of sSPECIAL (0.82) across regions. Taking all three test–rest reliability assessments together, our experiment proposes that MEGA‐sSPECIAL can be a more sensitive method with which to detect small changes in GABA in longitudinal and case‐control studies.

Evaluating the correlation between two measurement methods was proposed by Dhamala et al. as an alternative MRS validation method to complement the current use of CRLB and test–retest reproducibility evaluation because CRLB and reproducibility can show similar uncertainty estimation in a reproducible manner, but not the accuracy of the measurement method.^42^ In our results, sSPECIAL and MEGA‐sSPECIAL show overall strong correlations in the M1 and mPFC, and across the regions for NAA, Glu, GABA, and GABA/tCr, except for GABA and GABA/tCr in the M1 (Figure 8). The *r* correlation coefficients are comparable with values reported in the literature (NAA = 0.61–0.70; Glu = 0.85–0.87; GABA = 0.30–0.51).^42^ Furthermore, the homogeneous distribution of differences in Glu measurements indicates that there was no systematic difference between the two methods in the M1 (2.0%) and mPFC (2.6%). Regarding GABA measurements, even although differences are uniformly distributed, there were biases in GABA quantification of 51.0% in the M1 and 39.1% in the mPFC,^60^ which may have been induced by the systematic overestimation/underestimation of GABA by LCModel. The discrepancies in GABA concentrations between short‐TE and editing methods reported in other studies are presented in Table 4, where biases of 30% to 50% are observed.^11^

### 4.3 Reproducibility versus the number of averaged spectra

In a 3‐T study of between‐session reproducibility of GABA^+^ measurements by MEGA‐PRESS for 20‐min long acquisition, CVs were notably improved up to around 218 repetitions (approximately 13 min), and any further increase of the scanning time resulted in modest gains.^61^ In our study, the CVs were decreased by increasing the number of averages for both regions. However, unlike the 3‐T study, the CVs of GABA measurements by sSPECIAL at 7 T were not drastically diminished with the increased number of averaged spectra. With the increased number of averages from 32 to 128, the CVs of GABA reduced dramatically by 67% and 57% when using MEGA‐sSPECIAL in the M1 (from 12.5% to 4.1%) and mPFC (from 13.7% to 5.8%), respectively. However, the reduction in CVs using the sSPECIAL sequence is moderate, that is, 39% in the M1 (from 36.4% to 21.8%) and 33% in the mPFC (from 15.4% to 10.2%). As spectral dispersion and SNR both affect metabolite quantification, the moderate reduction in CV by the short‐TE method suggests that the spectral overlap even at 7 T remains a limiting factor for reliable quantification of GABA, regardless of the improvement in SNR.

In regions of low GABA concentrations such as M1, the editing method demonstrated substantially better reproducibility than short‐TE sSPECIAL with any number of averages. In such brain regions, spectral editing methods with resolved peak detection should be the method of choice. In a GABA‐rich region such as the mPFC, both short‐TE sSPECIAL and MEGA‐sSPECIAL showed good and comparable reproducibility, especially for averages of 32, 64, and 96, therefore short‐TE methods may be a favorable choice for these brain regions even with a small voxel size, as they are less sensitive to alterations in T_2_ relaxation times and offer simultaneously reliable quantification for other metabolites such as Gln, Lac, GSH, and Gly (CV < 15% in Table 3). Because the improvement in CVs is minor by increasing the number of averages using short‐TE sSPECIAL, a short measurement protocol with 64 averages could be used to reduce the measurement time by half (i.e., 4 min) without substantially affecting the reliability of GABA measurement. The CV values provided in our study can be used as guidance for protocol optimization in future clinical studies.

### 4.4 Limitations

Scheme 1 was chosen for in vivo comparison with the short‐TE method because it was the least sensitive method to frequency drift and B_1_ inhomogeneity in terms of GABA editing efficiency; however, it did not outperform the other schemes when taking the susceptibility of MM subtraction efficiency into account. Scheme 2 does not have coedited MMs, while the GABA editing efficacy is sensitive to both B_0_ and B_1_ inhomogeneity. The symmetric editing pulse application scheme (Scheme 3) has been extensively used to measure GABA without MM contamination,^22^, ^27^ and MM subtraction works efficiently under the on‐resonance condition.^62^ The main drawbacks of this method are the high sensitivity to frequency drift due to its dependence on symmetric editing pulse application and the degraded GABA efficiency due to B_1_ inhomogeneity.^17^ As the ground truth of metabolites in vivo is unknown, it is challenging to figure out the proportion of MM contamination in the acquired GABA signal by Scheme 3. The susceptibility to frequency drift of all three schemes can be reduced by the real‐time frequency update using the prospective motion correction and shim update.^63^ Future studies could be extended to evaluate the reproducibility of the other two editing schemes.

There is also a limitation in simulation and phantom experiments using Lys. Due to the complexity of in vivo MMs, Lys has been commonly used as a surrogate for coedited MM signal around 1.7 ppm.^32^ It is noteworthy that Lys does not fully represent MM contribution in in vivo spectra.

For statistical analyses to assess test–retest reliability and intersequence agreement, we used calculations of CV, Pearson's *r* correlation method, ICC evaluation, and the Bland–Altman plots together. However, considering the limited number of data samples and CV calculation using two scan sessions, the results should be interpreted with caution. The strength of correlations of GABA measurements was different for the mPFC and the M1. It was very strongly correlated in the mPFC but weakly inversely correlated in the M1, and this was similar to those measurements for GABA/tCr. In Dhamala et al.’s study,^42^ *r*‐values ranged from −0.11 to 0.37 for MEGA‐diff versus MEGA‐off, and from 0.42 to 0.51 for SPECIAL versus MEGA‐diff. There is a significant difference in reproducibility in the M1 between sSPECIAL and MEGA‐sSPECIAL. Therefore, the weak inverse correlation between GABA measurements in the M1 can be caused by a larger SD of values by sSPECIAL than that by MEGA‐diff.

Even although our study shows that GABA measurements using the editing method show better reproducibility than the short‐TE method, it has to be considered that four subspectra alignment is more sensitive to subject motion and frequency drift, especially for clinical applications. A single‐shot localization method may improve the susceptibility to motion. For example, the MEGA‐sLASER sequence was introduced using optimized TE to achieve improved editing efficiency and reduced chemical shift displacement error.^18^ Because it is based on a single‐shot localization sequence, in total two subspectra are used, which is less susceptible to motion artifacts.

In addition, it should be noted that the add‐subtract scheme of the editing method is more susceptible to artifacts from spurious echoes that originate from residual water in distant regions due to large magnetic field inhomogeneity outside the voxel, especially in applications using small voxels. Therefore, nonediting methods may outperform editing methods in these cases, which requires further validation in future studies.

## 5 CONCLUSIONS

MEGA‐sSPECIAL shows better reproducibility than short‐TE sSPECIAL in both the M1 and mPFC at 7 T. GABA measurements can be achieved reproducibly using both methods, even with low SNR in GABA‐rich regions, where short‐TE sSPECIAL can provide an assessment of full neurochemical profile simultaneously. In areas of low GABA concentration, the editing method is a more sensitive method to detect small changes in GABA concentration than the short‐TE method. T_2_ relaxation times should be estimated for accurate quantification, especially where relaxation times can be altered depending on pathological states, tissue types, and age.^45^ Lastly, the reproducibility obtained with the sSPECIAL and MEGA‐sSPECIAL sequences in multiregions at 7 T with in vivo‐measured neurochemical profiles can be used as guidance for setting up measurement protocols in future clinical applications.

## CONFLICT OF INTEREST

The authors declare that the research was performed in the absence of any commercial or financial relationships that could be construed as a potential conflict of interest.

## Supporting information


**Data S1.** Supporting Information

## Data Availability

The concentrations of metabolites and CRLBs used for all statistical analyses can be found under doi:10.5281/zenodo.5717118.
