# Supplementary material for: γ‐aminobutyric acid measurement in the human brain at 7 T: Short echo‐time or Mescher–Garwood editing
Source: NMR Biomed. 2022 Feb 18;35(7):e4706. doi: 10.1002/nbm.4706 (PMC9285498; doi:10.1002/nbm.4706)
Supplement: Supplementary file 1 — Data S1. Supporting Information [file NBM-35-0-s001.docx]

**Supplementary Material**

**Tables**

ST1: The metabolite T2 relaxation times used for correction

ST2: The mean MRS voxel placement and rotational angle with standard deviation in the mPFC and M1

ST1: The metabolite T2 relaxation times used for correction

| **Metabolite** | **T2 (ms)** | **Reference** |
| --- | --- | --- |
| Asp | 97 | ^1^Marjańska M, et al. |
| tCh | 139 | ^1^Marjańska M, et al. |
| tCr | 108 | ^1^Marjańska M, et al. |
| Glu | 98 | ^1^Marjańska M, et al. |
| Gln | 98 | ^1^Marjańska M, et al. |
| GABA | 87 | ^2^Adnreychenko A, et al. |
| GSH | 97 | ^1^Marjańska M, et al. |
| Ins | 100 | ^1^Marjańska M, et al. |
| Lac | 94 | ^3^Dehghani M, et al. |
| NAA | 110 | ^1^Marjańska M, et al. |
| NAAG | 110 | ^1^Marjańska M, et al. |
| Scyllo | 112 | ^1^Marjańska M, et al. |
| Tau | 90 | ^1^Marjańska M, et al. |
| PE | 90 | similar to Tau |
| Gly | 97 | similar to GSH |

1. Marjańska M, Auerbach EJ, Valabrègue R, Van de Moortele PF, Adriany G, Garwood M. Localized 1H NMR spectroscopy in different regions of human brain in vivo at 7T: T 2 relaxation times and concentrations of cerebral metabolites. NMR Biomed. 2012;25:332–339 doi: 10.1002/nbm.1754.

2. Andreychenko A, Klomp DWJ, De Graaf RA, Luijten PR, Boer VO. In vivo GABA T2 determination with J-refocused echo time extension at 7 T. NMR Biomed. 2013;26:1596–1601 doi: 10.1002/nbm.2997.

3. Dehghani M, Do KQ, Magistretti P, Xin L. Lactate measurement by neurochemical profiling in the dorsolateral prefrontal cortex at 7T: accuracy, precision, and relaxation times. Magn. Reson. Med. 2020;83:1895–1908 doi: 10.1002/mrm.28066.

ST2: The mean MRS voxel placement and rotational angle with standard deviation in the mPFC and M1

| **Regions** | **Session** | **Sagittal**  **(mm)** | **Coronal**  **(mm)** | **Transverse (mm)** | **Angle (°)** |
| --- | --- | --- | --- | --- | --- |
| **mPFC** | **1st** | 8.7 ± 2.3 | 46.8 ± 6.8 | 8.1 ± 5.9 | N/A |
| **mPFC** | **2nd** | 6.2 ± 3.5 | 48.8 ± 9.4 | 10.6 ± 5.9 | N/A |
| **M1** | **1st** | -29.7 ± 3.5 | -16.4 ± 3.9 | 19.7 ± 7.0 | 19 ± 3 |
| **M1** | **2nd** | -29.2 ± 1.9 | -15.2 ± 6.2 | 18.6 ± 7.3 | 22 ± 1 |
